# *Drosophila* phosphatidylinositol-4 kinase *fwd* promotes mitochondrial fission and can suppress *Pink1/parkin* phenotypes

**DOI:** 10.1371/journal.pgen.1008844

**Published:** 2020-10-21

**Authors:** Ana Terriente-Felix, Emma L. Wilson, Alexander J. Whitworth

**Affiliations:** 1 MRC Mitochondrial Biology Unit, University of Cambridge, Cambridge, United Kingdom; 2 Department of Biomedical Sciences, University of Sheffield, Sheffield, United Kingdom; Stanford University School of Medicine, UNITED STATES

## Abstract

Balanced mitochondrial fission and fusion play an important role in shaping and distributing mitochondria, as well as contributing to mitochondrial homeostasis and adaptation to stress. In particular, mitochondrial fission is required to facilitate degradation of damaged or dysfunctional units via mitophagy. Two Parkinson’s disease factors, PINK1 and Parkin, are considered key mediators of damage-induced mitophagy, and promoting mitochondrial fission is sufficient to suppress the pathological phenotypes in *Drosophila Pink1/parkin* mutants. We sought additional factors that impinge on mitochondrial dynamics and which may also suppress *Pink1/parkin* phenotypes. We found that the *Drosophila* phosphatidylinositol 4-kinase IIIβ homologue, Four wheel drive (Fwd), promotes mitochondrial fission downstream of the pro-fission factor Drp1. Previously described only as male sterile, we identified several new phenotypes in *fwd* mutants, including locomotor deficits and shortened lifespan, which are accompanied by mitochondrial dysfunction. Finally, we found that *fwd* overexpression can suppress locomotor deficits and mitochondrial disruption in *Pink1/parkin* mutants, consistent with its function in promoting mitochondrial fission. Together these results shed light on the complex mechanisms of mitochondrial fission and further underscore the potential of modulating mitochondrial fission/fusion dynamics in the context of neurodegeneration.

## Author summary

Mitochondria are dynamic organelles that can fuse and divide, in part to facilitate turnover of damaged components. These processes are essential to maintain a healthy mitochondrial network, and, in turn, sustain cell viability. This is critically important in high energy-demanding, post-mitotic tissues such as neurons. We previously identified *Drosophila* phosphatidylinositol-4 kinase *fwd* as a pro-fission factor in a cell-based screen. Here we show that loss of *fwd* regulates mitochondrial fission *in vivo*, and acts genetically downstream of *Drp1*. We identified new phenotypes in *fwd* mutants, similar to loss of *Pink1/parkin*, two genes linked to Parkinson’s disease and key regulators of mitochondrial homeostasis. Importantly, *fwd* overexpression is able to substantially suppress locomotor and mitochondrial phenotypes in *Pink1/parkin* mutants, suggesting that manipulating phosphoinositides may represent a route to tackling Parkinson’s disease.

## Introduction

Mitochondria are dynamic organelles that are transported to the extremities of the cell and frequently undergo fusion and fission events that influence their size, branching and degradation. Many of the core components of the mitochondrial fission and fusion machineries have been well characterised. There include the pro-fusion factors Mfn1/2 and Opa1, and pro-fission factors Drp1 and Mff [1]. Maintaining an appropriate balance of fission and fusion, as well as transport dynamics, is crucial for cellular health and survival as mutations in many of the core components cause severe neurological conditions in humans and model organisms [2]. Recently, a role for phosphatidylinositol 4-phosphate [PI(4)P] in mitochondrial fission has been elucidated in cultured cells [3], but the *in vivo* consequences have not yet been described.

The mitochondrial fission/fusion cycle has been linked to the selective removal of damaged mitochondria through the process of autophagy (termed mitophagy), in which defective mitochondria are engulfed into autophagosomes and degraded by lysosomes [4, 5]. Two genes that have been firmly linked to the mitophagy process are *PINK1* and *PRKN* [6–8]. Mutations in these genes cause autosomal-recessive juvenile parkinsonism, associated with degeneration of midbrain dopaminergic neurons and motor impairments, among other symptoms and pathologies. Studies from a wide variety of model systems have shown various degrees of mitochondrial dysfunction associated with mutation of *PINK1/PRKN* homologues including disrupted fission/fusion [9–17]. *Drosophila* have proven to be a fruitful model for investigating the function of the conserved homologues *Pink1* and *parkin*, with these mutants exhibiting robust mitochondrial disruption and neuromuscular phenotypes. Importantly, several studies have shown that the pathological consequences of loss of *Pink1* or *parkin* can be largely suppressed by genetic manipulations that increase mitochondrial fission or reduce fusion [18–24].

To identify genes involved in mitochondrial quality control and homeostasis, we previously performed an RNAi screen in *Drosophila* S2 cells to identify kinases and phosphatases that phenocopy or suppress hyperfused mitochondria caused by loss of *Pink1* [25]. We identified the phosphatidylinositol 4-kinase IIIβ homologue, *four wheel drive* (*fwd*), whose knockdown phenocopied *Pink1* RNAi, resulting in excess mitochondrial fusion. *Drosophila* mutant for *fwd* have been reported to be viable but male sterile due to incomplete cytokinesis during spermatogenesis [26–29]. While muscle-specific knockdown has shown to impact neuromuscular junction formation [30], no other organismal phenotypes or mitochondrial involvement have been described to date. Thus, we sought to better understand the role of Fwd in mitochondrial homeostasis.

In this study, we have characterised *fwd* mutants for organismal phenotypes associated with *Pink1/parkin* dysfunction, and analysed the impact on mitochondrial form and function. We have also investigated genetic interactions between *fwd* and *Pink1/parkin*, as well as with mitochondrial fission/fusion factors. We found that loss of *fwd* inhibited mitochondrial function, causing increased mitochondrial length and branching, and decreased respiratory capacity. These effects were associated with shortened lifespan and dramatically reduced locomotor ability, similar to *Pink1* and *parkin* mutants. Furthermore, *fwd* overexpression was sufficient to significantly suppress *Pink1/parkin* mutant locomotor deficits and mitochondrial phenotypes. Interestingly, we found that the mitochondrial and locomotion phenotypes in *fwd* mutants can be rescued by loss of pro-fusion factors *Marf* and *Opa1*, but the pro-fission activity of *Drp1* appears to require *fwd*. These results support a role for *fwd* in regulating mitochondrial morphology, specifically in facilitating mitochondrial fission, and further substantiate the important contribution of aberrant mitochondrial fission/fusion dynamics in *Pink1/parkin* phenotypes.

## Results

### Loss of *fwd* causes mitochondrial hyperfusion along with locomotor and lifespan deficits

We previously found that knockdown of *fwd* phenocopied loss of *Pink1* in cultured cells by causing mitochondrial hyperfusion [25]. To extend these *in vitro* observations we sought to determine whether *fwd* has a broader role in regulating mitochondrial homeostasis *in vivo*. In striking similarity to *Pink1* mutants, mutations in *fwd* have previously been shown to cause male sterility due to aberrant spermatogenesis [26–29]; however, no other organismal phenotypes have been described for these mutants.

**Fig 1:**
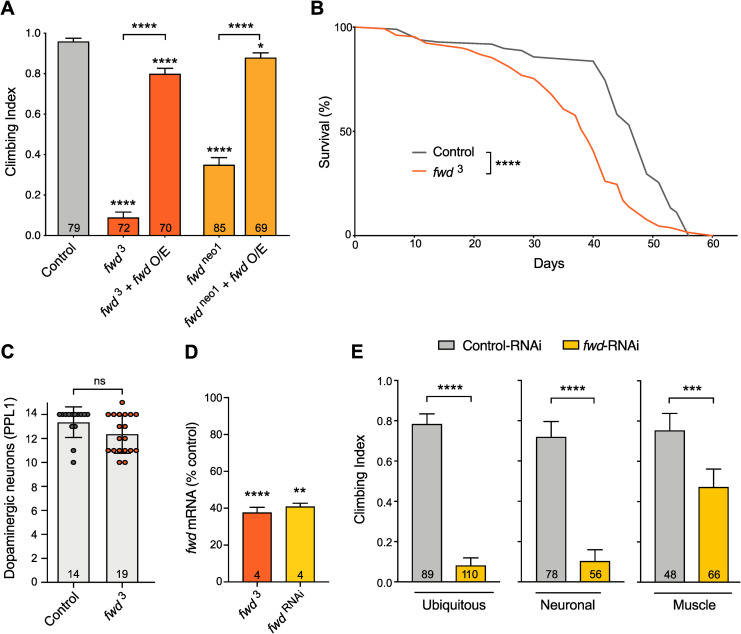
Loss of *fwd* causes motor deficits and shortened lifespan. (A) Climbing assay of *fwd* mutants (*fwd*^3^ and *fwd*^neo1^) in trans to a deficiency (Df), alone or with transgenic overexpression of *fwd* (*fwd* O/E) driven by *da*-*GAL4*. (B) Lifespan analysis of control and *fwd* mutants. Significance for lifespan was analysed by log-rank (Mantel-Cox) test. (C) Quantification of dopaminergic neurons in PPL1 cluster of 30-day-old adult brains. Chart shows mean ± SD with individual data points. Significance was analysed by Mann-Whitney test. (D) Expression analysis of *fwd* transcript levels in adults of the *fwd*^3^ mutant and RNAi driven by *da*-*GAL4*. Charts show mean ± SEM. Significance was analysed by unpaired *t*-test against their respective controls; number of biological replicates is shown in each bar. (E) Climbing analysis of *fwd* knockdown (RNAi) in all (ubiquitous) or selected tissues. Climbing assays charts in (A) and (E) show mean ± 95% confidence interval (CI); number of animals analysed is shown in each bar. Significance for climbing was analysed by Kruskal-Wallis test with Dunn’s post-hoc correction for multiple comparisons. Comparison is against the control unless otherwise indicated; * *P<*0.05, ** *P<*0.01, *** *P<*0.001, **** *P<*0.0001; ns, non-significant. Full genotypes are given in S1 Table.

*Pink1* mutants have a range of additional phenotypes including deficits in negative geotaxis (climbing ability), disruption of flight muscles, shortened lifespan, and modest degeneration of dopaminergic (DA) neurons [11,31], all of them associated with disrupted mitochondrial morphology and function. Thus, we assessed these phenotypes in two *fwd* mutants–a nonsense mutation, *fwd*^3^, and a complex chromosomal rearrangement, *fwd*^neo1^ (also called *fwd*^1^ [28]). In all instances, these mutations were crossed to a deficiency (*Df(3L)7C*) to avoid potential extragenic effects from homozygosity. Both mutant combinations, *fwd*^3^/Df and *fwd*^neo1^/Df (hereafter, designated simply as *fwd*^3^ and *fwd*^neo1^), displayed a striking loss of climbing ability in young flies (Fig 1A), though the phenotype was weaker in *fwd*^neo1^ consistent with it being a hypomorph. Notably, transgenic re-expression of *fwd* using a ubiquitous driver (*da*-*GAL4*), was able to restore climbing ability to near wild-type levels (Fig 1A), supporting the specificity of this phenotype for loss of *fwd*. Analysing longevity in the *fwd*^3^ null mutants revealed a significant reduction in median lifespan (Fig 1B). However, no significant loss of DA neurons was detected in aged *fwd* mutant brains (Fig 1C). These results reveal some phenotypic similarity between *Pink1* and *fwd* mutants at the organismal level as well as the cellular level.

To investigate the relative contribution of *fwd* to locomotor ability in different tissues, we expressed a transgenic RNAi construct [32] via tissue-specific drivers. We first verified that ubiquitous knockdown of *fwd* via *da*-*GAL4* phenocopied the genetic mutants in terms of loss of transcripts (Fig 1D) and recapitulation of the climbing phenotype (Fig 1E). Interestingly, pan-neuronal knockdown, using *nSyb-GAL4*, reproduced the striking loss of climbing ability, whereas knockdown in all muscles via *Mef2-GAL4* only modestly affected climbing (Fig 1E). Thus, *fwd* shows some tissue-selective requirement but plays an important role in the nervous system that was not previously appreciated.

**Fig 2:**
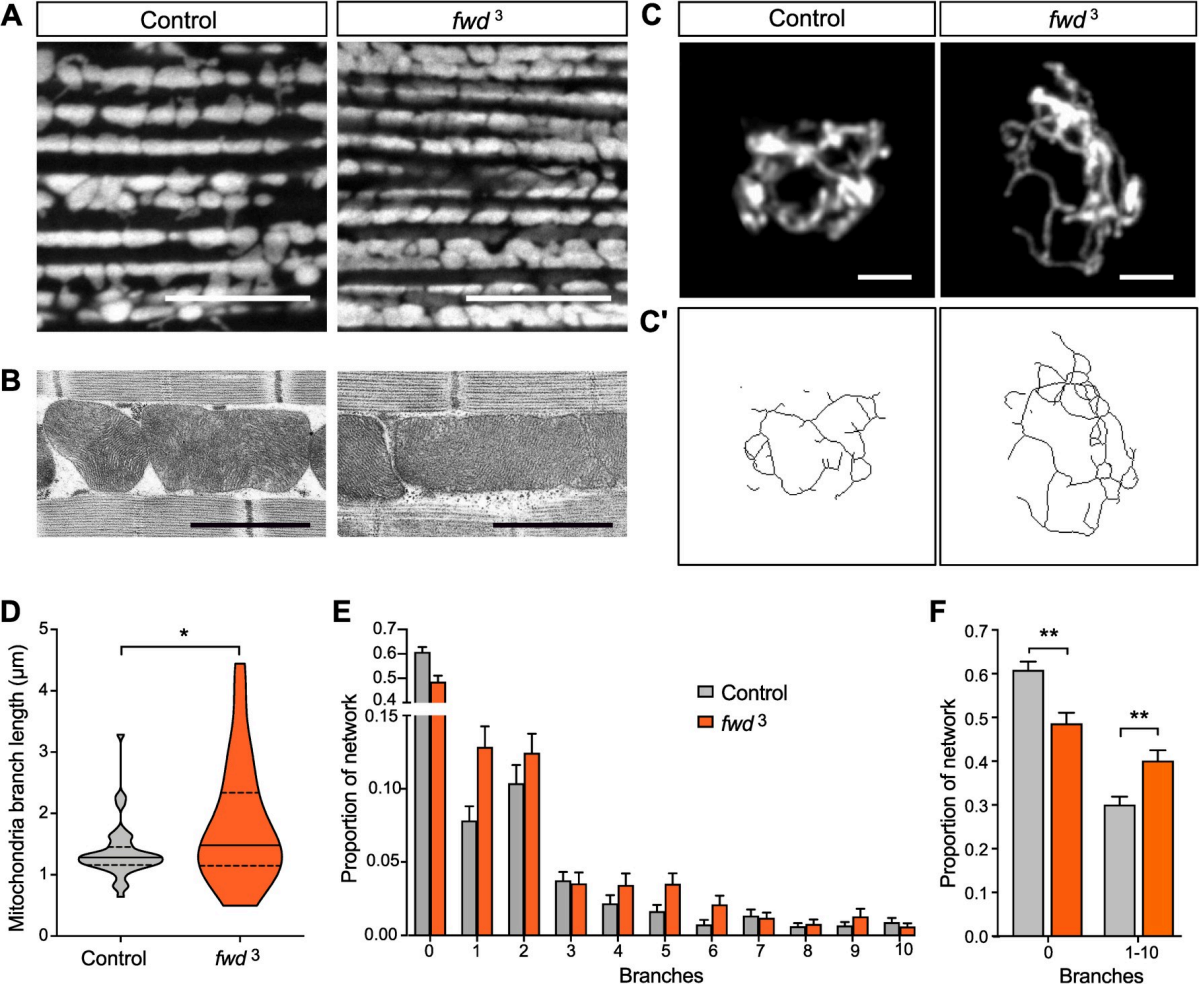
Loss of *fwd* causes excess mitochondrial fusion. (A) Confocal microscopy analysis of mitochondrial morphology, visualised using mitoGFP, in control and *fwd* mutant flight muscles. Scale bar = 10 μm. (B) Electron-microscopy analysis of mitochondrial structure in flight muscles. Scale bar = 1 μm. (C) Confocal microscopy analysis of mitochondrial network morphology (mitoGFP) in neuronal cell bodies from larval ventral ganglion of control and *fwd* mutants. Image shows a projected z-stack. Scale bar = 2 μm. (C’) Skeletonised image of mitochondrial network used for quantification. (D) Quantification of median mitochondrial branch length per cell. Violin plot indicating median (thick horizontal line) and quartiles (dashed lines). Significance was analysed by Mann-Whitney test. (E) Frequency distribution plot of mitochondrial network connectivity (number of connected branches) per cell. N = 46 (Control) and 54 (*fwd*^3^). (F) Chart summarising quantification of connectivity shown in E, plotting the proportion of individual mitochondria (0 branches) and connected mitochondria (1–10 branches) relative to the total number of networks per cell. Significance was analysed by Kruskal-Wallis test with Dunn’s post-hoc correction for multiple comparisons. * *P<*0.05, ** *P<*0.01. Full genotypes are given in S1 Table.

Since *fwd* knockdown in cultured cells caused mitochondrial fusion, similar to loss of *Pink1*, we sought to further characterise the impact of *fwd* loss on mitochondria *in vivo*. Mitochondria are particularly abundant in adult flight muscles, and this tissue is severely affected in *Pink1/parkin* mutants [9,11,31], so we first analysed mitochondrial morphology in *fwd* mutants in this tissue. Imaging mitochondria by fluorescence or electron-microscopy in flight muscles revealed them to be grossly normal in their cristae structure, size, and abundance, compared to control (Fig 2A and 2B).

We next sought to analyse the mitochondrial morphology in a tissue where the specific knockdown of *fwd* resulted in strong climbing defects. We analysed the network morphology in cell bodies of the larval ventral ganglion (part of the central nervous system). Expression of mitoGFP in a subset of neurons, driven by *CCAP*-*GAL4*, allowed better three-dimensional imaging of the mitochondrial network (Fig 2C). While the overall appearance was similar between *fwd* mutant and control, quantitative analysis of the networks revealed that both the length and connectivity (number of branches) were increased upon loss of *fwd* (Fig 2D–2F). These results are consistent with the previous cell-based study indicating that loss of *fwd* causes mitochondrial hyperfusion.

**Fig 3:**
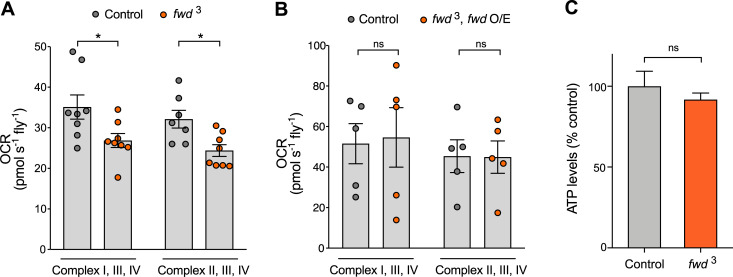
Loss of *fwd* inhibits mitochondrial respiration. (A, B) Mitochondrial respiration analysis by oxygen consumption rate (OCR) in control and *fwd* mutant adults, and *fwd* mutant ubiquitously re-expressing *fwd*. (C) ATP levels in control and *fwd* mutant adults. Charts show mean ± SEM. Significance was analysed by paired (A, B) or unpaired (C) *t*-test. * *P<*0.05; ns, non-significant. Full genotypes are given in S1 Table.

We next assessed mitochondrial function, analysing maximal respiratory capacity in intact mitochondria and overall ATP levels in whole animals. Respiration measured by the oxygen consumption rate in energised mitochondria, stimulated via either complex I or complex II substrates, was significantly reduced in *fwd* mutants (Fig 3A), and was completely rescued by *fwd* re-expression (Fig 3B). However, the overall level of ATP was not significantly affected (Fig 3C). These results indicate that mitochondrial respiration is affected by loss of *fwd* but compensatory mechanisms could still maintain normal steady-state ATP levels in the organism.

### *fwd* mutant phenotypes are suppressed by loss of fusion factors

**Fig 4:**
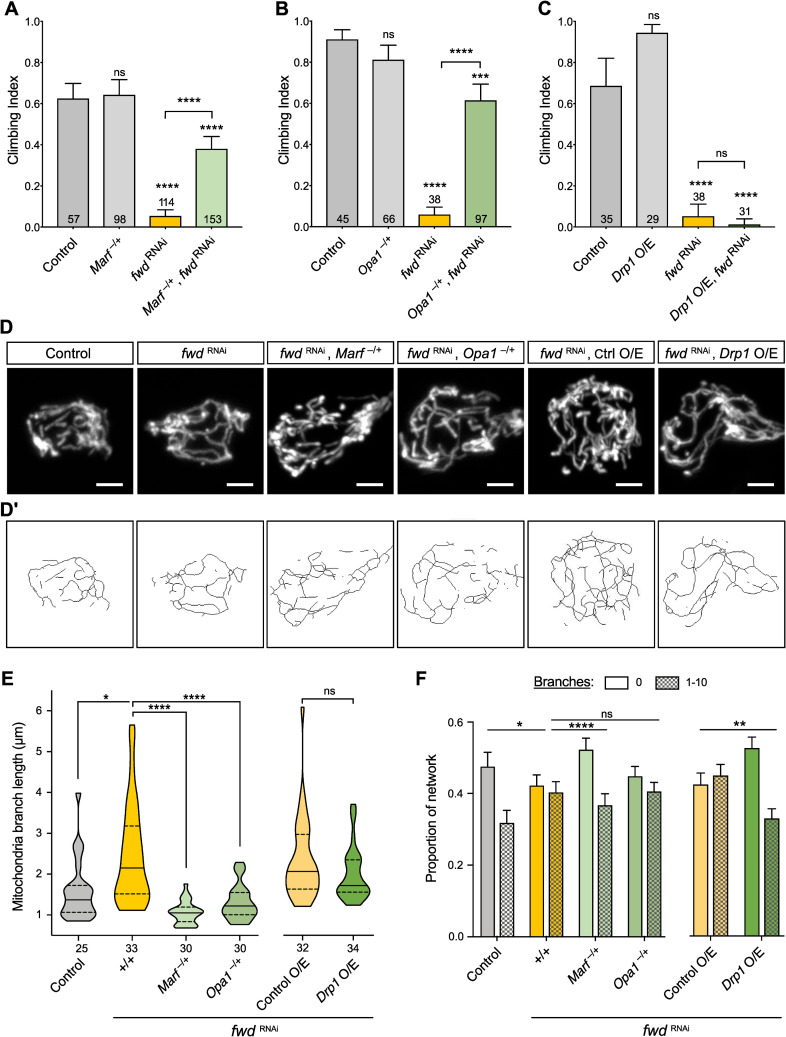
*fwd* genetically interacts with mitochondrial fission/fusion factors. (A-C) Climbing assay of *fwd* RNAi alone or in combination with heterozygous *Marf* or *Opa1* mutations or transgenic overexpression of *Drp1*. Transgenic expression was mediated via *da-GAL4*. Charts show mean ± 95% confidence interval (CI); number of animals analysed is shown in each bar. (D) Confocal microscopy analysis of mitochondrial network morphology (mitoGFP) in neuronal cell bodies from larval ventral ganglion of control, *fwd* RNAi alone and in combination with heterozygous *Marf* or *Opa1* mutations or transgenic overexpression of *Drp1*. Image shows a projected z-stack. Scale bar = 2 μm. (D’) Skeletonised image of mitochondrial network used for quantification. (E) Quantification of median mitochondrial branch length per cell. Violin plot indicating median (solid horizontal line) and quartiles (dashed lines). Significance was analysed by Mann-Whitney test (left) or unpaired *t*-test (right). The number of cells analysed is indicated below each plot. (F) Plot of the proportion of individual mitochondria (0 branches) and connected mitochondria (1–10 branches) quantified per cell. Significance was calculated by Kruskal-Wallis test with Dunn’s post-hoc correction for multiple comparisons (left) or *t*-test (right). Comparison is against the control unless otherwise indicated; * *P<*0.05, ** *P*<0.01, *** *P<*0.001, **** *P<*0.0001; ns, non-significant. Full genotypes are given in S1 Table.

The results above substantiate that loss of *fwd* causes excess mitochondrial fusion *in vivo*. We next addressed whether the mitochondrial hyperfusion may contribute to the locomotor deficit. To do this we combined ubiquitous expression of *fwd* RNAi with genetic manipulations that reduce fusion (partial loss of pro-fusion factors *Marf* or *Opa1*) or promote fission (overexpression of pro-fission factor *Drp1*), and assessed climbing behaviour. Heterozygous loss of either *Marf* (the fly homologue of *MFN1/2*) or *Opa1*, which did not affect climbing alone, was sufficient to significantly suppress the climbing deficit caused by *fwd* RNAi (Fig 4A and 4B). However, contrary to what we expected, overexpression of *Drp1* was not able to ameliorate the climbing defect (Fig 4C). Importantly, knockdown of *fwd* did not affect the levels of endogenous or overexpressed *Drp1* (S2 Fig), indicating that the lack of suppression was not due to insufficient expression.

To better understand these results, we analysed the mitochondrial morphology in neuronal cell bodies of these genotypes. As with the *fwd* mutant, *fwd* RNAi caused a significant elongation of mitochondria and increased branching (Fig 4D–4F). Consistent with the effects on climbing, heterozygous loss of *Marf* or *Opa1* reverted the increase in mitochondrial length, whereas *Drp1* overexpression did not (Fig 4D and 4E). Interestingly, the increased branching caused by loss of *fwd* was suppressed by heterozygous loss of *Marf* or *Drp1* overexpression, but not by heterozygous loss of *Opa1* (Fig 4D and 4F). The reasons for the complex effects on branching are unclear but may reflect that Marf directs fusion of the outer mitochondrial membrane and hence, coordinates branching, while Opa1 regulates fusion of inner mitochondrial membrane. Nevertheless, the effects on mitochondrial branch length suggest that Drp1 may require Fwd to execute mitochondrial fission. Overall, the genetic interaction of *Marf* and *Opa1* suppressing the *fwd* RNAi-induced climbing deficit supports this phenotype being, at least partially, caused by mitochondrial hyperfusion.

### *fwd* overexpression can suppress *Pink1/parkin* mutant phenotypes

**Fig 5:**
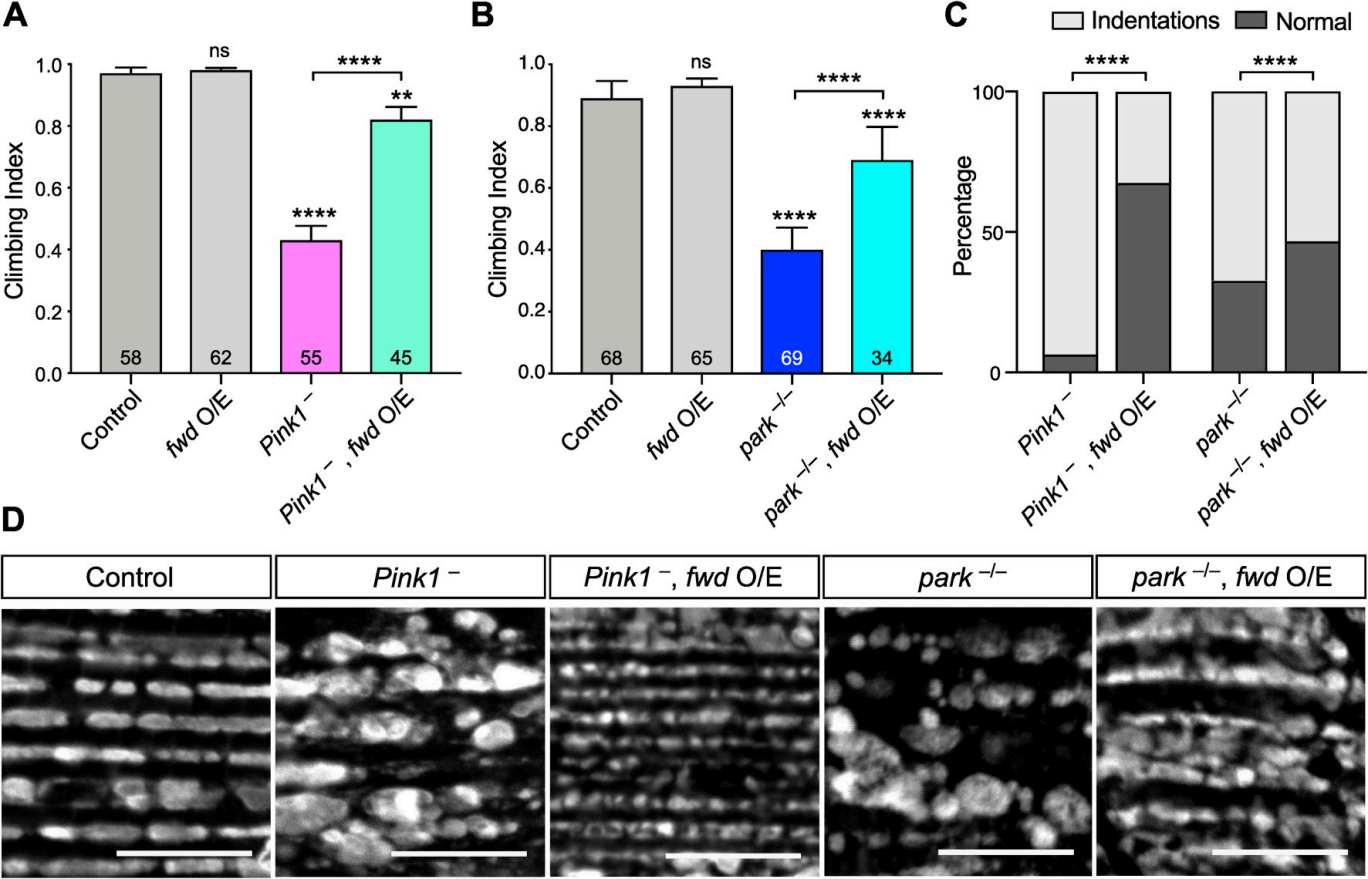
*fwd* overexpression partially suppresses *Pink1/parkin* phenotypes. (A, B) Climbing assay of control, *Pink1* or *parkin* mutants with or without ubiquitous *fwd* overexpression. Charts show mean ± 95% confidence interval (CI); number of animals analysed is shown in each bar. Significance was analysed by Kruskal-Wallis test with Dunn’s post-hoc correction for multiple comparisons. Comparison is against the control unless otherwise indicated; ** *P*<0.01, **** *P<*0.0001; ns, non-significant. (C) Analysis of thoracic indentations evident in *Pink1* or *parkin* mutants in the presence or absence of *fwd* overexpression, induced by *da-GAL4*. Significance was determined by Chi-squared test. **** *P<*0.0001. (D) Confocal microscopy analysis of mitochondrial integrity, visualised by anti-ATP5A immunostaining, in flight muscles of the indicated genotypes. Transgenic expression was mediated via *Mef2-GAL4*. Scale bar = 10 μm. Full genotypes are given in S1 Table.

While many studies have focused on the role of PINK1/Parkin in damage-induced mitophagy, aberrant mitochondrial dynamics is clearly a major cause of *Pink1/parkin* mutant phenotypes in *Drosophila*, including locomotor deficits and flight muscle degeneration, since these can be substantially suppressed by promoting mitochondrial fission [18–21]. As our results indicate that Fwd promotes mitochondrial fission, we next tested whether overexpression of *fwd* could ameliorate *Pink1* and *parkin* mutant phenotypes. Combining *Pink1/parkin* mutants with ubiquitous *fwd* overexpression was sufficient to significantly suppress the climbing deficit in both mutants (Fig 5A and 5B). In addition, the thoracic indentations caused by degeneration of the underlying flight muscle were also significantly corrected (Fig 5C). Disruption of mitochondrial integrity in the flight muscles was also visibly improved when *fwd* was overexpressed in muscles (Fig 5D). These results are consistent with Fwd overexpression promoting mitochondrial fission and partially reverting the hyperfusion caused by *Pink1* and *parkin* loss.

**Fig 6:**
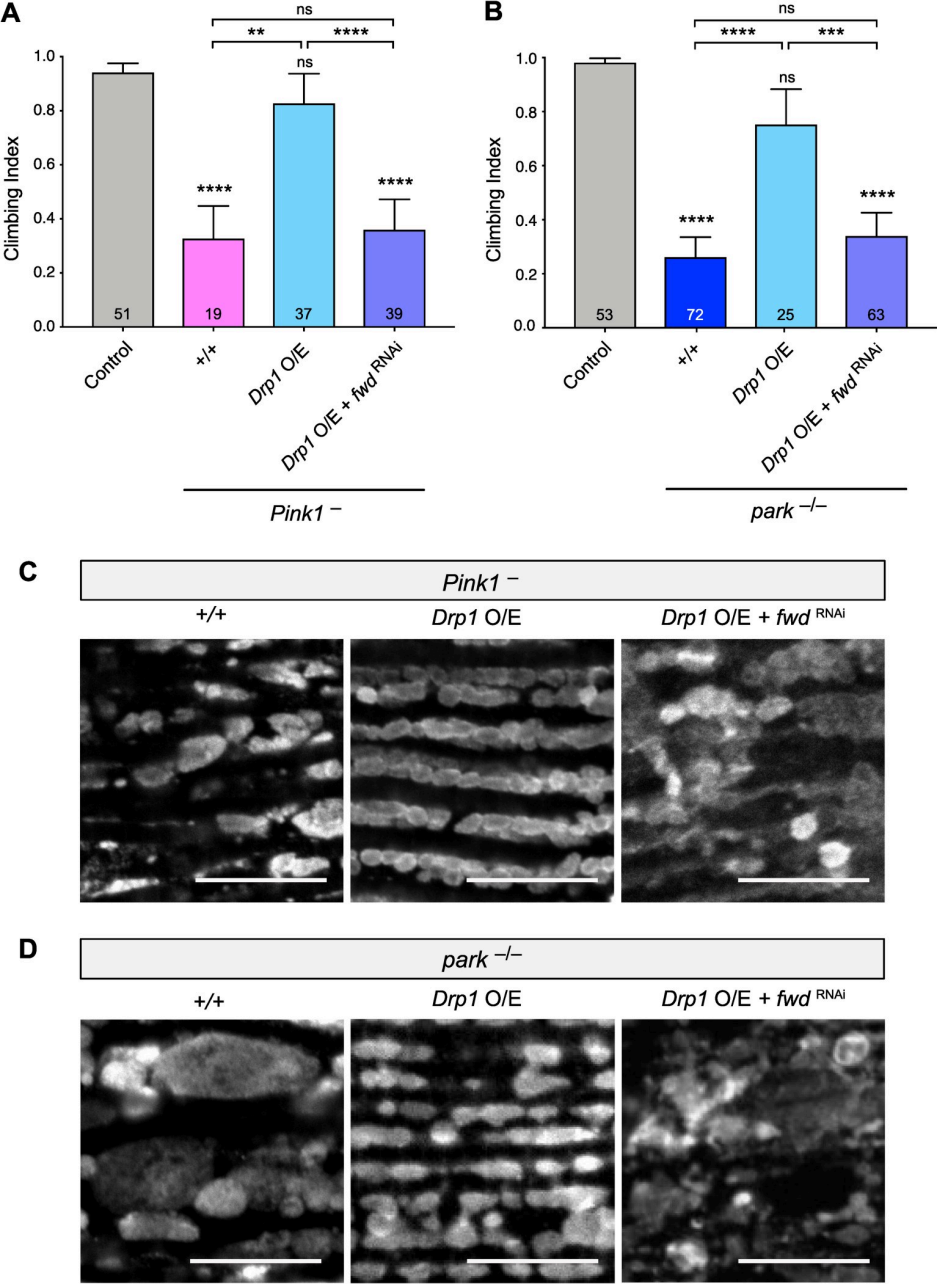
*Drp1* activity requires *fwd* in suppressing *Pink1/parkin* phenotypes. (A, B) Climbing assay of control, *Pink1* or *parkin* mutants with or without *Drp1* overexpression or concomitant induction of *fwd* RNAi. Significance was analysed by Kruskal-Wallis test with Dunn’s post-hoc correction for multiple comparisons. Comparison is against the control unless otherwise indicated; ** *P<*0.01, *** *P<*0.001, **** *P<*0.0001; ns, non-significant. (C, D) Confocal microscopy analysis of mitochondrial integrity, visualised by anti-ATP5A immunostaining, in flight muscles of the indicated genotypes. For all conditions, transgenic expression was mediated via *da-GAL4*. Scale bar = 10 μm. Full genotypes are given in S1 Table.

We were intrigued by the earlier observation that heterozygous loss of *Marf* or *Opa1* could revert the increased mitochondrial length and climbing defect of *fwd* RNAi, but the overexpression of *Drp1* did not (Fig 4). These results suggested that the activity of Drp1 might require Fwd, which we sought to test further. As a paradigm for Drp1 activity, overexpression of *Drp1* is sufficient to substantially suppress the climbing deficit and mitochondrial disruption in *Pink1* and *parkin* mutants (Fig 6A–6D), as previously reported [19]. Remarkably, coincident knockdown of *fwd* completely prevented the ability of Drp1 to rescue the *Pink1/parkin* mutant phenotypes (Fig 6A–6D). These results further indicate that Drp1 requires the activity of Fwd.

## Discussion

We previously identified *fwd* as a gene whose knockdown induces mitochondrial hyperfusion in cultured cells, similar to loss of *Pink1* [25]. Here we have validated that the genetic loss or knockdown of *fwd* also causes excess mitochondrial fusion in neuronal cells *in vivo*, leading to increased mitochondrial length and branching. As mitochondrial fission/fusion dynamics have been shown to be important for mitochondrial homeostasis [2], it is not surprising that this also has an impact on respiration at the organismal level, and on organismal fitness and vitality. While *fwd* mutants have mainly been characterised for their male sterility phenotype, we describe new organismal phenotypes associated with loss of *fwd*: profound locomotor deficits and shortened lifespan. Interestingly, while our data reveal a stronger requirement for *fwd* in the nervous system compared to the musculature to maintain normal motor behaviour, *fwd* is required in muscle for neuromuscular junction formation [30]. Furthermore, consistent with our observations on lifespan, Fwd overexpression has previously been shown to confer increased lifespan [33]. Thus, Fwd clearly has a more widespread role in organismal vitality than previously appreciated.

The robust locomotor phenotype allowed us to test the genetic relationship between *fwd* and core components of the mitochondrial fission/fusion machinery. Given the excess mitochondrial fusion upon loss of *fwd*, suppression of the organismal phenotypes by reduction of fusion factors *Marf* and *Opa1* was expected. However, it was surprising that overexpression of the fission factor *Drp1* was unable to ameliorate organismal phenotypes or even the increased mitochondrial length, though it was able to revert the increased branching caused by loss of *fwd*. These results suggested that Drp1 requires Fwd to drive mitochondrial fission. Consistent with this, *Drp1* overexpression was no longer able to rescue *Pink1/parkin* mutant phenotypes in the absence of *fwd*. These genetic experiments strongly hint at a functional link between Drp1 and Fwd but do not illuminate the molecular mechanism underpinning it. Fwd is the *Drosophila* homologue of phosphatidylinositol 4-kinase IIIβ [PI(4)KB], which mediates the phosphorylation of phosphatidylinositol to generate phosphatidylinositol 4-phosphate [PI(4)P] [34]. PI(4)P is one of the most abundant phosphoinositides, which is usually concentrated in the trans-Golgi network [35]; thus, the mechanism by which PI(4)P may influence mitochondrial dynamics is not immediately obvious. However, while this manuscript was in preparation, Nagashima and colleagues reported that Golgi-derived PI(4)P-containing vesicles were required for the final stages of mitochondrial fission [3]. In that study, the authors found that loss of PI(4)KIIIβ led to hyperfusion and increased branching of the mitochondrial network, consistent with what we observed here. Moreover, they described that while Drp1 was still recruited, it was unable to fully execute the scission event, although the reason is unclear, leading to extended mitochondrial constriction sites. Our genetic evidence that the action of Drp1 requires Fwd is consistent with these findings, and provide an *in vivo* validation of Nagashima and colleagues’ results. Currently, it is unclear why *Drp1* overexpression was able to revert the increased branching caused by loss of *fwd* but the mechanisms of branch formations are not well understood. It is interesting to note that while Nagashima et al. suggest a universal role for PI(4)P in mitochondrial fission, our *in vivo* analysis reveals that while *fwd* affected mitochondrial morphology in the nervous system, it appeared to have a much more limited role in the musculature. These tissue-specific requirements were borne out in the strong locomotor deficits caused by neuronal loss of *fwd* but much less so by knockdown in muscles. Clearly, further work is required to better understand the complexities of regulated fission/fusion events in different cell contexts *in vivo*.

A key role of mitochondrial fission/fusion dynamics is in contributing to a quality control mechanism of mitochondrial sorting to eliminate dysfunctional units via mitophagy [4,5]. A substantial body of evidence from cellular models indicates that mammalian PINK1 and Parkin act to promote damage-induced mitophagy [6–8], and some *in vivo* evidence from *Drosophila* also supports this [36,37]. However, the precise nature of PINK1/Parkin-mediated mitochondrial turnover *in vivo* is debated with contradictory results emerging [38–42]. Nevertheless, interventions to combat the decline in mitochondrial homeostasis remain a key challenge to combatting *PINK1/PRKN* related pathologies. One mechanism that seems to provide substantial benefit in physiological contexts is through augmenting mitochondrial fission, which presumably facilitates the flux of damaged mitochondrial components towards turnover [18–21]. Here, we provide further evidence that augmenting a pro-fission pathway is beneficial against *Pink1* and *parkin* dysfunction. As phosphoinositides can be interconverted by the action of multiple enzymes that may be druggable, these findings suggest another potential route towards a therapeutic intervention.

## Methods

### *Drosophila* stocks and husbandry

Flies were raised and kept under standard conditions in a temperature-controlled incubator with a 12h:12h light:dark cycle at 25°C and 65% relative humidity, on food consisting of agar, cornmeal, molasses, propionic acid and yeast. The following strains were obtained from the Bloomington *Drosophila* Stock Center (RRID:SCR_006457): *w*^1118^ (RRID:BDSC_6326), *fwd*^neo1^ (RRID:BDSC_10069), *Df(3L)7C* (RRID:BDSC_5837), *Opa1*^s3475^ (RRID:BDSC_12188), *da-GAL4* (RRID:BDSC_55850), *nSyb-GAL4* (RRID:BDSC_51941), *Mef2-GAL4* (RRID:BDSC_27390), *CCAP-GAL4* (RRID:BDSC_25685, RRID:BDSC_25686), UAS*-mito-HA-GFP* (RRID:BDSC_8442, RRID:BDSC_8443), UAS*-fwd*^RNAi^ (RRID:BDSC_35257), UAS-*Luciferase*^RNAi^ (RRID:BDSC_31603). Other lines were kindly provided as follows: *fwd*^3^ from J. Brill [28], and the *Pink1*^B9^ and UAS-*Drp1* from J. Chung [11], *Marf*^B^ from H. Bellen [43], UAS*-mito-mCherry* from A. Vagnoni [44]. The *park*^25^ mutants have been described previously [9]. UAS-*GFP-fwd* was generated by PCR amplification of the GFP-fwd sequence from a *hsp83*::*GFP-fwd* plasmid [29], kindly provided by G. Polevoy and J. Brill, and cloned into pUAST.attB for integration at the attP40 locus (BestGene Inc.). All experiments in adult flies were conducted using males, except Fig 4A where females were used.

### Locomotor assays

The repetitive iteration startle-induced negative geotaxis (RISING, or ‘climbing’) assay was performed using a counter-current apparatus. Experiments were performed using 2–3 day old flies. Except for Fig 4A, all the climbing assays used males. Briefly, 20–23 flies were placed into the first chamber, tapped to the bottom, and given 10 s to climb a 10 cm distance. This procedure was repeated five times (five chambers), and the number of flies that remained in each chamber counted. The weighted performance of several groups of flies for each genotype was normalized to the maximum possible score and expressed as *Climbing index* [9].

### Lifespan

For lifespan experiments, flies were grown under identical conditions at low density. Progeny were collected under very light anaesthesia (i.e. as little time as possible on CO_2_ to sort genotypes) and kept in tubes of approximately 25 males each. Flies were transferred every 2–3 days to fresh media and the number of dead flies recorded. Percent survival was calculated at the end of the experiment after correcting for any accidental loss.

### Immunohistochemistry and sample preparation

For immunostaining of muscle mitochondria, adult flight muscles were dissected and fixed in PBS-4% formaldehyde for 30 min at RT, permeabilized in PBS-0.3% Triton X-100 for 30 min, and blocked with PBS-0.3% Triton X-100 plus 4% Horse Serum (HS) for 1 h at RT. Tissues were incubated with ATP5A antibody (Abcam Cat# ab14748, RRID:AB_301447; 1:500), diluted in PBS-0.3% Triton X-100 plus 4% HS overnight at 4°C, then rinsed 3 times 10 min with PBS-0.3% Triton X-100, and incubated with the appropriate fluorescent secondary antibodies overnight at 4°C. The tissues were washed twice in PBS and mounted on slides. For immunostaining of dopaminergic neurons, adult brains were dissected in PBS and fixed on ice in PBS-4% formaldehyde for 30 min, permeabilized in PBS-0.3% Triton X-100 for 30 min, and blocked with PBS-0.3% Triton X-100 plus 4% HS for 4 h at RT. Incubation with Tyrosine Hydroxylase Antibody (TH) antibody (Inmunostar Cat#22941, 1:200) diluted in PBS-0.3% Triton X-100 plus 4% HS was done for 72h at 4°C. Secondary antibody was incubated for 3 h at RT. Then washes were done 3 times for 20 min with PBS-0.3% Triton X-100 and mounted in carved slides with posterior facing the coverslip. For immunostaining of CCAP neurons, larval brains were dissected in PBS and mounted sideways inside a silicone rubber well formed on a slide coated with poly-lysine at 0.9 mg/ml. They were fixed in PBS-4% formaldehyde for 20 min at RT, washed in PBS, then the silicone well was removed with a scalpel. All the sample preparations were mounted using Prolong Diamond Antifade mounting medium (Thermo Fischer Scientific Cat# P36961).

### Microscopy

Fluorescence imaging was conducted using a Zeiss LSM 880 confocal microscope (Carl Zeiss MicroImaging) equipped with Plan-Apochromat 100x/1.4 Oil DIC M27 immersion objective. Images were taken at a resolution of 2048x2048 pixels, speed 7 and averaging 4, and were processed using Fiji software (RRID:SCR_002285).

### Analysis of mitochondrial morphology

The expression of the mitochondrial marker mitoGFP by *CCAP*-*GAL4* driver was analysed in cell bodies from larvae ventral nerve cord. All images were processed using Fiji software (RRID:SCR_002285). Z-stacks of individual neurons were cropped. The mitoGFP signal was enhanced and smoothed using two filters: unsharp mask (radius = 10.0 pixels, Mask strength 0.9) and median filtering (radius = 3). The binary masks were created using Image>Adjust>Threshold., Method:Otsu, and Background:Dark. Then, branches were generated using Process>Binary>Skeletonised. These skeletonised images were analysed using Analyse>Skeleton>Analyse Skeleton (2D/3D). Examples of skeletonised images of individual planes from a z-stack are shown in Supporting Information (S1 Fig). Median branch length per cell was calculated using “Branch Length” column from "Branch information" window, and the proportion of individual vs interconnected branches per cell was calculated by taking the “Number of branches” column from the “Results” window.

### Transmission electron-microscopy

Thoraces were prepared from 5-day-old adult flies and treated as previously described [9]. Ultra-thin sections were examined using a FEI Tecnai G2 Spirit 120KV transmission electron-microscope.

### Respirometry analysis

Respiration was monitored at 30 ^o^C using an Oxygraph-2k high-resolution respirometer (OROBOROS Instruments) using a chamber volume of 2 mL. Calibration with air-saturated medium was performed at the beginning of every session. Data acquisition and analysis were carried out using Datlab software (OROBOROS Instruments). Five flies per genotype (equal weight) were homogenised in MiR05 respiration buffer. For coupled (state 3) assays, complex I-linked respiration was measured at saturating concentrations of malate (2 mM), glutamate (10 mM) and adenosine diphosphate (ADP, 2.5 mM). Prior to complex II-linked respiration assay 0.15 μM rotenone was added, then 10 mM succinate was supplemented. Data from 7–8 independent experiments were averaged.

### ATP levels

The ATP assay was performed as described previously [25]. Briefly, five male flies for each genotype were homogenized in 100 μL 6 M guanidine-Tris/EDTA extraction buffer and subjected to rapid freezing in liquid nitrogen. Homogenates were diluted 1/100 in the extraction buffer and mixed with the luminescent solution (CellTiter-Glo Luminescent Cell Viability Assay, Promega RRID:SCR_006724, Cat. #G7571). Luminescence was measured with a SpectraMax Gemini XPS luminometer (Molecular Devices). The average luminescent signal from technical triplicates was expressed relative to protein levels, quantified using the Pierce BCA Protein Assay kit (ThermoFisher Scientific, RRID: SCR_008452, Cat. #23227). Data from 3 independent experiments were averaged and the luminescence expressed as a percentage of the control.

### qRT-PCR expression analysis

Total RNA from 3 adult males or 6 larvae per biological replicate was extracted using TRI Reagent(R) (Merck Life Science UK Limited, Sigma-Aldrich, RRID:SCR_008988, Cat. #T9424). 300 ng of total RNA was used for reverse transcription with Maxima H Minus cDNA Synthesis Master Mix with dsDNAse (Thermo Fisher Scientific, RRID: SCR_008452, Cat. #M1681). Quantitative real-time PCR (qRT-PCR) was performed on a QuantStudio 3 Real-Time PCR Systems (Thermo Fisher Scientific, RRID:SCR_008452) with Maxima SYBR Green/ROX qPCR Master Mix (Thermo Fisher Scientific, RRID: SCR_008452, Cat. #K0221). Primers were as follows: (*fwd*) 5’-CTTGGGATTCGAACAGTCGC-3’ and 5’-TGCTGCGCATAATTTCCACGAA-3’, (α*Tub84b*) 5′-TGGGCCCGTCTGGACCACAA and 5′-TCGCCGTCACCGGAGTCCAT-3’. Primers’ efficiency was between 0.92 and 0.94. Non-RT control was used to assess any residual genomic DNA contamination. The relative transcript levels of each target gene were normalized against α*Tub84b* mRNA levels; quantification was performed using the comparative C_T_ method taking into account the efficiency of each primer pair [45].

### Statistical analysis

Data from the various experimental assays were analysed as follows: For behavioural analyses, Kruskal-Wallis non-parametric test with Dunn’s post-hoc correction for multiple comparisons was used. Lifespan was analysed by Log-rank (Mantel-Cox) test. Categorical analyses (i.e. thoracic indentations) were analysed by Chi-square test. Mitochondrial branch length by Mann-Whitney non-parametric test or unpaired *t*-test between pairs, and connectivity by Kruskal-Wallis with Dunn’s post-hoc correction or unpaired *t*-test between pairs. Expression levels were analysed by unpaired *t*-test or one-way ANOVA with Sidak’s correction. ATP levels were analysed by unpaired *t*-test, and respiration by paired *t*-test. Analyses were performed using GraphPad Prism 8 software (RRID:SCR_002798) and RStudio software (RRID:SCR_000432).

## Supporting information

S1 Fig Examples of skeletonised images for mitochondrial morphology analysis. (PDF)

S2 Fig Analysis of *Drp1* transcript levels upon *fwd* RNAi. Quantitative real-time PCR analysis of *Drp1* transcript levels under basal conditions in adults (A) or *Drp1* overexpression in larvae (B). Charts show mean ± SEM. Significance was analysed by unpaired *t*-test (A) or ANOVA with Sidak’s correction (B) against their respective controls (see S1 Table); **** *P<*0.0001; ns, non-significant; number of biological replicates is shown in each bar.
(PDF)

S1 Table Full genotypes of animals used in all figures. (DOCX)
