# Supplementary figures and images for: Drosophila phosphatidylinositol-4 kinase fwd promotes mitochondrial fission and can suppress Pink1/parkin phenotypes
Source: PLoS Genet. 2020 Oct 21;16(10):e1008844. doi: 10.1371/journal.pgen.1008844 (PMC7605714; doi:10.1371/journal.pgen.1008844)

# **A** From Figure 2C

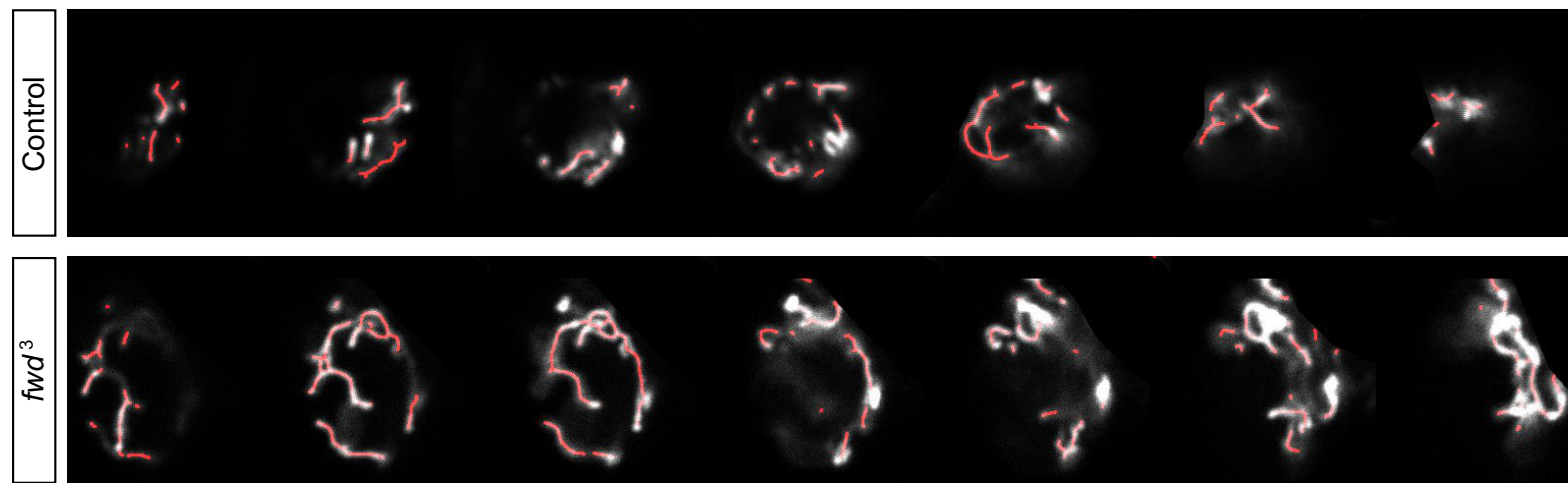

# **B** From Figure 4D

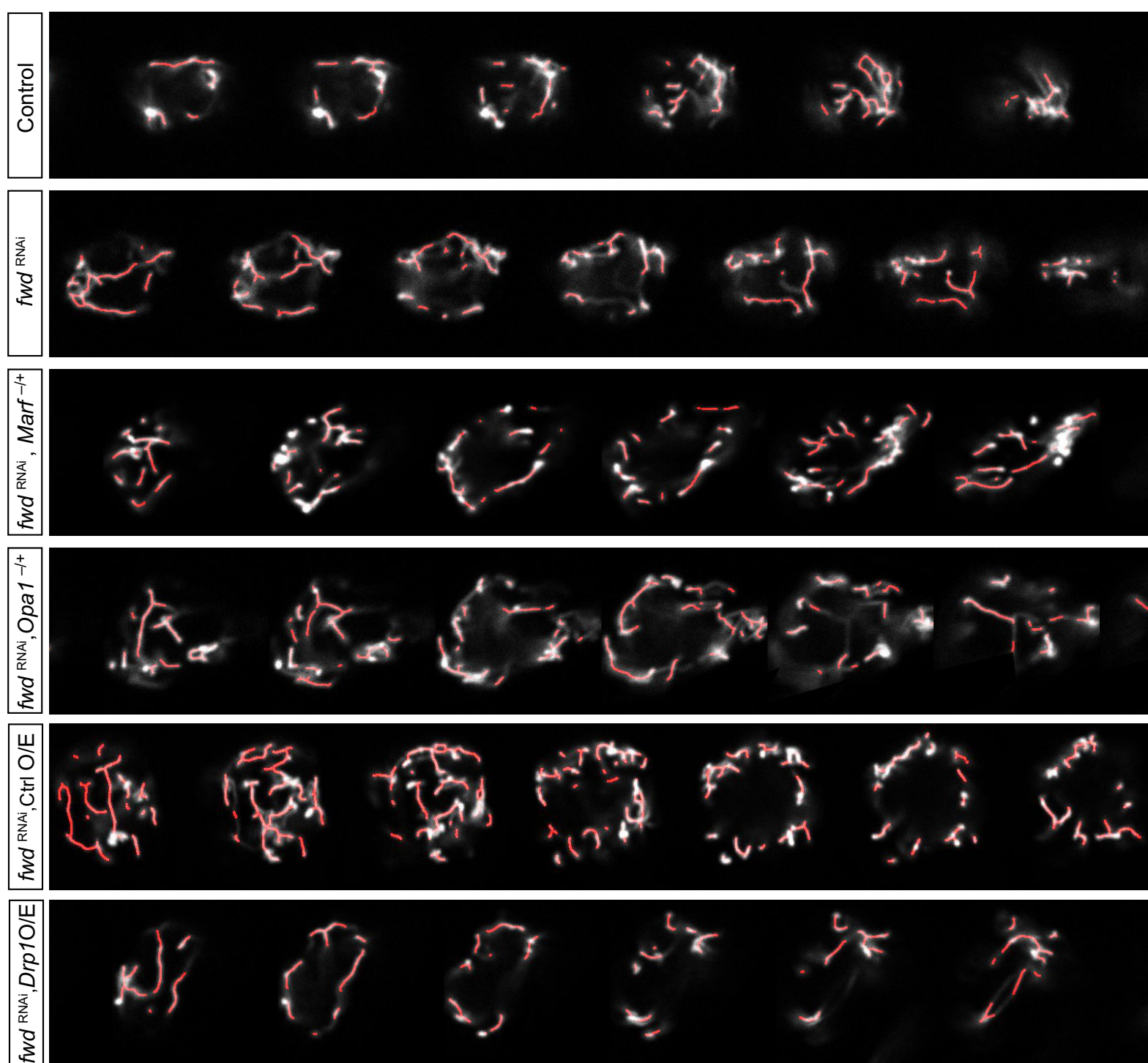

Supplement: S1 Fig — (PDF) [file pgen.1008844.s001.pdf]

**A**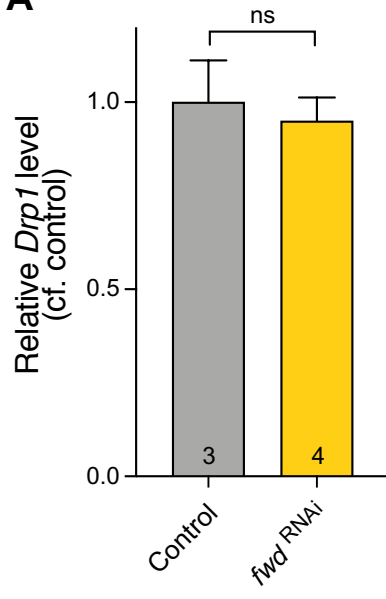**B**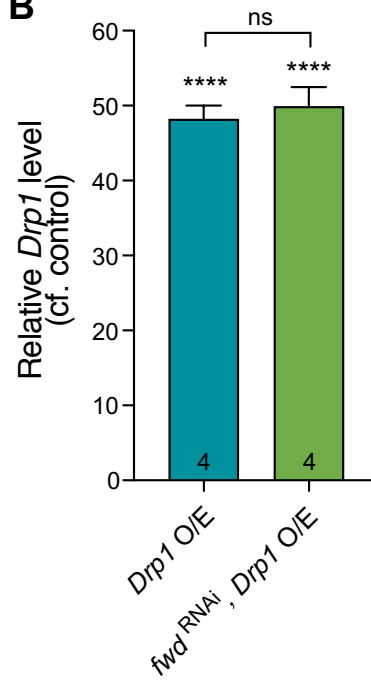

Supplement: S2 Fig — Quantitative real-time PCR analysis of Drp1 transcript levels under basal conditions in adults (A) or Drp1 overexpression in larvae (B). Charts show mean ± SEM. Significance was analysed by unpaired t-test (A) or ANOVA with Sidak’s correction (B) against their respective controls (see S1 Table); **** P<0.0001; ns, non-significant; number of biological replicates is shown in each bar. (PDF) [file pgen.1008844.s002.pdf]
