# Supplementary material for: Drosophila phosphatidylinositol-4 kinase fwd promotes mitochondrial fission and can suppress Pink1/parkin phenotypes
Source: PLoS Genet. 2020 Oct 21;16(10):e1008844. doi: 10.1371/journal.pgen.1008844 (PMC7605714; doi:10.1371/journal.pgen.1008844)
Supplement: S1 Table — (DOCX) [file pgen.1008844.s003.docx]

**Supplementary Table 1**. Details of full genotypes used in this study. More details of each line can be found in Methods.

| **Figure 1** | |
| --- | --- |
| **Label** | **Genotype** |
| **A** | |
| Control | da-GAL4/+ |
| fwd^3^ | fwd^3^/Df(3L)7C, da-GAL4 |
| fwd^3^ + fwd O/E | UAS-GFP-fwd/+; fwd^3^/Df(3L)7C, da-GAL4 |
| fwd^neo1^ | fwd^neo1^/Df(3L)7C, da-GAL4 |
| fwd^neo1^ + fwd O/E | UAS-GFP-fwd/+; fwd^neo1^/Df(3L)7C, da-GAL4 |
| **B** | |
| Control | da-GAL4/+ |
| fwd^3^ | fwd^3^/Df(3L)7C, da-GAL4 |
| **C** | |
| Control | da-GAL4/+ |
| fwd^3^ | fwd^3^/Df(3L)7C |
| **D** | |
| Control (not shown) | w^1118^ |
| fwd^3^ | fwd^3^/Df(3L)7C |
| Control (not shown) | da-GAL4/+ |
| fwd^RNAi^ | da-GAL4/UAS-fwd-RNAi |
| **E** | |
| Ubiquitous: | |
| control-RNAi | da-GAL4/UAS-Luciferase-RNAi |
| fwd-RNAi | da-GAL4/UAS-fwd-RNAi |
| Neuronal: | |
| control-RNAi | nSyb-GAL4/UAS-Luciferase-RNAi |
| fwd-RNAi | nSyb-GAL4/UAS-fwd-RNAi |
| Muscle: | |
| control-RNAi | Mef2-GAL4/UAS-Luciferase-RNAi |
| fwd-RNAi | Mef2-GAL4/UAS-fwd-RNAi |

| **Figure 2** | | |
| --- | --- | --- |
| **Label** | **Genotype** | |
| **A** | | |
| Control | UAS-mito-HA-GFP/+; da-GAL4/+ |  |
| fwd^3^ | UAS-mito-HA-GFP/+; fwd^3^/Df(3L)7C, da-GAL4 |  |
| **B** | |  |
| Control | da-GAL4/+ |  |
| fwd^3^ | fwd^3^/Df(3L)7C, da-GAL4 |  |
| **C-F** | |  |
| Control | CCAP-GAL4,UAS-mito-Tomato/ UAS-mito-HA-GFP; fwd^3^/+ |  |
| fwd^3^ | CCAP-GAL4,UAS-mito-Tomato/UAS-mito-HA-GFP; fwd^3^/Df(3L)7C |  |

| **Figure 3** | | |
| --- | --- | --- |
| **Label** | **Genotype** | |
| **A, C** | |  |
| Control | da-GAL4/+ |  |
| fwd^3^ | fwd^3^/Df(3L)7C, da-GAL4 |  |
| **B** | |  |
| Control | da-GAL4/+ |  |
| fwd^3^ | UAS-GFP-fwd/+; fwd^3^/Df(3L)7C, da-GAL4 |  |

| **Figure 4** | | |
| --- | --- | --- |
| **Label** | **Genotype** | |
| **A** | |  |
| Control | da-GAL4/UAS-mito-HA-GFP |  |
| Marf ^–/+^ | Marf^B^/w^1118^; da-GAL4/ UAS-mito-HA-GFP |  |
| fwd^RNAi^ | da-GAL4/UAS-fwd-RNAi |  |
| Marf ^–/+^; fwd^RNAi^ | Marf^B^/ w^1118^; da-GAL4/UAS-fwd-RNAi |  |
| **B** | |  |
| Control | da-GAL4/ UAS-mito-HA-GFP |  |
| Opa1^–/+^ | Opa1^s3475^/+; da-GAL4/UAS-mito-HA-GFP |  |
| fwd^RNAi^ | da-GAL4/UAS-fwd-RNAi |  |
| Opa1^–/+^; fwd^RNAi^ | Opa1^s3475^/+; da-GAL4/UAS-fwd-RNAi |  |
| **C** | |  |
| Control | da-GAL4/+ |  |
| Drp1 O/E | UAS-mito-HA-GFP/+; da-GAL4/UAS-Drp1 |  |
| fwd^RNAi^ | UAS-mito-HA-GFP/+; da-GAL4/UAS-fwd-RNAi |  |
| Drp1 O/E + fwd^RNAi^ | da-GAL4/UAS-fwd-RNAi, UAS-Drp1 |  |
| **D, E, F** | |  |
| Control | UAS-mito-HA-GFP/+; CCAP-GAL4/+ |  |
| fwd^RNAi^ | UAS-mito-HA-GFP/+; CCAP-GAL4/UAS-fwd-RNAi |  |
| fwd^RNAi^, Marf ^–/+^ | Marf^B^/w^1118^; UAS-mito-HA-GFP/+; CCAP-GAL4/UAS-fwd-RNAi |  |
| fwd^RNAi^, Opa1^–/+^ | UAS-mito-HA-GFP/Opa1^s3475^; CCAP-GAL4/UAS-fwd-RNAi |  |
| fwd^RNAi^, Ctrl O/E | UAS-mito-HA-GFP/UAS-mito-mCherry; CCAP-GAL4/UAS-fwd-RNAi |  |
| fwd^RNAi^, Drp1 O/E | UAS-mito-HA-GFP/+; CCAP-GAL4/UAS-fwd-RNAi, UAS-Drp1 |  |

| **Figure 5** | | |
| --- | --- | --- |
| **Label** | **Genotype** | |
| **A, C** | |  |
| Control | da-GAL4/+ |  |
| fwd O/E | UAS-GFP-fwd/+; da-GAL4/+ |  |
| Pink1^–^ | Pink1^B9^/Y; da-GAL4/+ |  |
| Pink1^–^, fwd O/E | Pink1^B9^/Y; UAS-GFP-fwd/+; da-GAL4/+ |  |
| **B, C** | |  |
| Control | da-GAL4/+ |  |
| fwd O/E | UAS-GFP-fwd/+; da-GAL4/+ |  |
| park^–/–^ | park^25^/ park^25^, da-GAL4 |  |
| park^–/–^, fwd O/E | UAS-GFP-fwd/+; park^25^/park^25^, da-GAL4 |  |
| **D** | |  |
| Control | UAS-mito-HA-GFP/+; Mef2-GAL4/+ |  |
| Pink1^–^ | Pink1^B9^/Y; UAS-mito-HA-GFP/+; Mef2-GAL4/+ |  |
| Pink1^–^, fwd O/E | Pink1^B9^/Y; UAS-GFP-fwd/+; Mef2-GAL4/+ |  |
| park^–/–^ | park^25^/ park^25^, Mef2-GAL4 |  |
| park^–/–^, fwd O/E | UAS-GFP-fwd/+; park^25^/ park^25^, Mef2-GAL4 |  |

| **Figure 6** | | |
| --- | --- | --- |
| **Label** | **Genotype** | |
| **A, C** | | |
| Control | UAS-mito-HA-GFP /+; da-GAL4/+ |  |
| Pink1^–^ | Pink1^B9^/Y; UAS-mito-HA-GFP/+; da-GAL4/+ |  |
| Pink1^–^, Drp1 O/E | Pink1^B9^/Y; UAS-mito-HA-GFP/+; da-GAL4/UAS-Drp1 |  |
| Pink1^–^, Drp1 O/E + fwd^RNAi^ | Pink1^B9^/Y; da-GAL4/UAS-fwd-RNAi, UAS-Drp1 |  |
| **B, D** | |  |
| Control | UAS-mito-HA-GFP /+; da-GAL4/+ |  |
| park^–/–^ | park^25^/park^25^, da-GAL4 |  |
| park^–/–^, Drp1 O/E | UAS-mito-HA-GFP/+; park^25^, UAS-Drp1/park^25^, da-GAL4 |  |
| park^–/–^, Drp1 O/E + fwd^RNAi^ | UAS-fwd-RNAi, park^25^, UAS-Drp1/park^25^, da-GAL4 |  |

| **S2 Figure** | | |
| --- | --- | --- |
| **Label** | **Genotype** | |
| **A** | |  |
| Control | da-GAL4/UAS-Luciferase-RNAi |  |
| fwd^RNAi^ | da-GAL4/UAS-fwd-RNAi |  |
| **B** | |  |
| Control (not shown) | da-GAL4/UAS-Luciferase-RNAi |  |
| Drp1 O/E | da-GAL4/ UAS-Drp1 |  |
| Drp1 O/E + fwd^RNAi^ | da-GAL4/UAS-fwd-RNAi, UAS-Drp1 |  |
